# Cryopreservation of primary neonatal rat oligodendrocytes and recapitulation of *in vitro* oligodendrocyte characteristics

**DOI:** 10.3389/fncel.2024.1520992

**Published:** 2025-01-13

**Authors:** Hanki Kim, Bum Jun Kim, Seungyon Koh, Hyo Jin Cho, Byung Gon Kim, Jun Young Choi

**Affiliations:** ^1^Department of Brain Science, Ajou University School of Medicine, Suwon, Republic of Korea; ^2^Department of Biomedical Sciences, Ajou University Graduate School of Medicine, Suwon, Republic of Korea; ^3^Department of Neurology, Ajou University School of Medicine, Suwon, Republic of Korea

**Keywords:** oligodendrocyte, oligodendrocyte progenitor cell (OPC), primary culture, cryopreservation, *in vitro* myelination

## Abstract

**Introduction:**

*In vitro*, primary rat oligodendrocytes (OLs) are widely used for research on OL development, physiology, and pathophysiology in demyelinating diseases such as multiple sclerosis. Primary culture methods for OLs from rats have been developed and improved over time, but there are still multiple aspects in which efficiency can be boosted.

**Methods:**

To make use of excess oligodendrocyte progenitor cells (OPCs) from primary cultures, a cryopreservation process utilizing a commercially available serum-free cryopreservation medium was established to passage and freeze OPCs at −80°C for later use.

**Results:**

Cryopreserved OPCs stored for up to 6 months were viable, and retained their OL lineage purity of ~98%. While OPCs cryopreserved for 3–6 months showed a decrease in cell density after two days of proliferation, ~17% of cryopreserved OPCs maintained the potential for proliferation comparable to control OPCs that had not frozen. After induction of differentiation for four days, ~43% of both control and cryopreserved OPCs differentiated into mature OLs, and when differentiation was induced on aligned nanofibers mimicking axonal structure, myelin sheath-like structures indicative of *in vitro* myelination was observed in all experimental groups.

**Conclusion:**

The validation of cryopreserved primary OLs as a functionally robust *in vitro* model can help improve the efficiency of primary OL culture, expand its applications, and reduce the inevitable sacrifice of animals.

## 1 Introduction

Oligodendrocytes (OLs) are central nervous system (CNS) glia, responsible for myelination, saltatory conduction, and metabolic support to myelinated neurons (Bradl and Lassmann, 2010; Philips and Rothstein, 2017). OL characteristics and pathology have been extensively studied, both *in vivo* and *in vitro*. *In vivo* animal models hold the advantage of preserved signals, microenvironments, and interactions. *In vitro* cell culture models provide a simplified environment in which OLs can be isolated from other brain cells, and core functions of OLs, such as oligodendrocyte progenitor cell (OPC) proliferation, migration, and differentiation, can be induced using specific growth factors and signals (Collarini et al., 1991; Baron et al., 2000; Barres et al., 1994). A large bulk of *in vitro* research on OLs has been performed with primary OLs derived from neonatal rats, embryonic stem cells, and induced pluripotent stem cells. Various methods for primary OL culture, such as the “shaking” method, immunopanning, and magnetic-associated cell sorting, have been devised and widely used (McCarthy and de Vellis, 1980; Emery and Dugas, 2017; Weil et al., 2019).

Recently, the authors developed a new method to isolate and cultivate primary OLs from neonatal rodent brains, which we have coined the E3 (easy, efficient, and effective) method, allowing for the acquisition of large quantities of highly pure OPCs and excels in its simplicity of procedure, minimal requirements, and cost-effectiveness (Kim et al., 2024). The high yield of the E3 method led to an excess of OPCs, and to make use of this excess, we applied cell cryopreservation. The cryopreservation of cultured OPCs helps reduce the sacrifice of animals, and also enables certain applications such as OPC transplantation, which has been demonstrated as a viable option to facilitate remyelination in animal models of demyelinating diseases.

In the present article, we introduce the method for cryopreservation of primary rat OPCs cultured on two-dimensional surfaces and report the extent to which these cryopreserved OPCs could recapitulate cardinal *in vitro* OL functions such as OPC proliferation, OL differentiation, and *in vitro* myelination.

## 2 Materials and methods

### 2.1 Animals

The Institutional Animal Care and Use Committee of Ajou University School of Medicine reviewed and approved all animal experiments, which complied with the National Institutes of Health (NIH) Guide for the Care and Use of Laboratory Animals. Postnatal day 1 (P1) Sprague–Dawley (SD) rats were purchased from Orient Bio Korea, Inc.

### 2.2 Passaging and cryopreservation of OPC cultures

OPCs were isolated from P1 SD rat cerebral cortices through a two-step differential centrifugation process developed and previously reported by the authors (Kim et al., 2024). The isolated OPCs were seeded onto Poly-D-Lysine (PDL, Gibco, #A3890401)-coated T75 flasks (Corning, #430641 U) and proliferated for 5 days with OPC proliferation media: Dulbecco’s Modified Eagle Medium/Ham’s F12 (DMEM/F12, Thermo Fisher Scientific, #11320033) with 1% GlutaMAX (Thermo Fisher Scientific, #35050-061), 1% penicillin/streptomycin (Cytiva, #SV30010), 2% B-27 supplement (Thermo Fisher Scientific, #17504-044), 30 ng/mL platelet-derived growth factor-AA (PDGF-AA, PeproTech, #100-13A), 10 ng/mL fibroblast growth factor (FGF, PeproTech, #100-18B), 10 ng/mL epidermal growth factor (EGF, PeproTech, #AF-100-15) (Kim et al., 2024).

After 5 days of proliferation, confluent colonies of OPCs were observed. The cells were detached with 5 mL/flask of Accutase (Thermo Fisher Scientific, #A11105-01) and pelleted by centrifugation, 200 × g/3 min/room temperature (RT = ~23°C). For the direct subculture of passaged OPCs, the cell pellet was resuspended in 1 mL of OPC stabilization media (=OPC proliferation media minus EGF), counted with a dual-fluorescence cell counter, seeded onto PDL-coated 96-well plates or 9 mm coverslips (SPL, #20009) at 1 × 10^4^ live cells/cm^2^, and maintained for 2 days. For the cryopreservation of passaged OPCs, the cell pellet was resuspended in 2 mL/flask CELLBANKER 2 (Zenogen Pharma, Japan), a serum-free cryopreservation medium, and counted with a dual-fluorescence cell counter (Logos Biosystems, #L20001) utilizing acridine orange/propridium iodide (AO/PI). The cell suspension in CELLBANKER 2 was transferred to cryopreservation vials (Thermo Fisher Scientific, #368632), two vials/flask, 1 mL/vial, and the vials were placed in a cell cryopreservation container (Thermo Fisher Scientific, #5100-0001) utilizing isopropanol. The containers were placed in a −80°C deep freezer, as recommended by the manufacturer, for 1 day, after which the cryovials were taken out of the container and stored at −80°C for 1–6 months. On the day of usage, the vial to be used was thawed in a 37°C water bath, and the thawed OPCs in CELLBANKER 2 were transferred to a 15 mL conical tube. Two milliliters of phosphate-buffered saline (PBS) was added, and the tube was centrifuged at 200 × g/3 min/RT. The pellet was resuspended in 1 mL of OPC stabilization media, and the cell yield and viability were measured with a dual fluorescence cell counter. The thawed OPCs were plated on PDL-coated 96-well plates or 9 mm coverslips at 1 × 10^4^ live cells/cm^2^ in OPC stabilization media and maintained for 2 days. Experimental groups used throughout the article were: (1) control (OPCs that were not frozen), (2) 1–2 months cryopreserved OPCs, (3) 3–6 months cryopreserved OPCs.

### 2.3 OL differentiation and *in vitro* myelination on aligned nanofibers

After 2 days of maintenance in OPC stabilization media, control and cryopreserved OPCs underwent differentiation for 4 days by exchange of culture media to OL differentiation media: Neurobasal medium (NBM, Thermo Fisher Scientific, #21103049) with 1% GlutaMAX, 1% penicillin/streptomycin, 2% B-27, 1% N2 supplement (Thermo Fisher Scientific, #17502048) and 40 ng/mL triiodothyronine (thyroid hormone T3, Sigma Aldrich, #T6397) (Kim et al., 2024). For *in vitro* myelination, differentiation was induced on OPCs seeded onto PDL-coated cell culture inserts with aligned nanofibers (Sigma Aldrich, #Z694614-12EA) (Kim et al., 2024; Bechler, 2019).

### 2.4 Immunofluorescence

At the endpoint of OPC stabilization or OL differentiation, cells were fixed with 4% paraformaldehyde for 20 min at RT and stored at 4°C until staining. Blocking was performed for 1 h at RT, with a blocking solution comprising 10% normal goat serum (NGS) and 0.1% Triton X-100 in PBS. Following a PBS wash, the cells were incubated overnight (~16 h) at 4°C with the following primary antibodies suspended in the blocking solution: anti-NG2 (1:200, Merck Millipore, #AB5320), anti-myelin basic protein (MBP) (1:500, Abcam, #AB7349), anti-Olig2 (1:200, Merck Millipore, #MABN50), anti-adenomatous polyposis coli (APC-CC1) (1:200, Merck Millipore, #OP80). The cells were washed with PBS, and secondary antibody incubation was conducted for 1 h at RT, with goat secondary antibodies conjugated with Alexa Fluor 488, 594, and 680 (Thermo Fisher Scientific). Cell nuclei were counterstained with 4′,6-diamidino-2-phenylindole, dihydrochloride (DAPI, 1 μg/mL in distilled water, Sigma Aldrich, #D9542) for 10 min at RT, and the cells were finally mounted onto glass slides. A Zeiss LSM800 confocal microscope was used to visualize the results.

### 2.5 EdU proliferation assay

An EdU Assay/EdU Staining Proliferation Kit (iFluor 488) (Abcam, #ab219801) was used to assess OPC proliferation, and EdU staining was performed according to the manufacturer’s protocol. Control and cryopreserved OPCs were stabilized/proliferated for 2 days, and before sample collection and fixation, the OPCs were incubated with EdU (1:1000) for 1 h. The cells were immunostained for Olig2 according to the steps described in section 2.4, with an additional step for EdU labeling with a reaction mixture containing iFluor 488 azide, 30 min at RT, after secondary antibody incubation and before cell nuclei counterstaining.

### 2.6 Statistics and image quantification

Statistical analyses used in the article were conducted with GraphPad Prism, version 10.3.0 (GraphPad Software, Boston, Massachusetts, United States). One-way analysis of variance (ANOVA), Kruskal–Wallis tests with Dunn’s multiple comparison tests, and simple linear regression were performed. Statistical analyses were two-sided, and statistical significance was defined as a *p-*value of <0.05. Cell counting and positive cell detection were performed with the *Positive Cell Detection* function in QuPath (Bankhead et al., 2017). Fractal dimension analysis to quantify cell morphological complexity was performed with the box counting method by using the *Fractal Box Count* function in Fiji/ImageJ (Schindelin et al., 2012). The equations used for image quantification were as such: OL purity = Olig2^+^ & DAPI^+^ cells/DAPI^+^ cells, OPC proliferation (cell count) = Olig2^+^ cells per mm^2^ (measured from 16 images, total 3.17mm^2^ and containing ~1,200 OPCs, per sample), OPC proliferation (EdU) = EdU^+^ & Olig2^+^ cells/Olig2^+^ cells, mature OL proportion = MBP^+^ & DAPI^+^ cells/DAPI^+^ cells, APC-CC1^+^ & DAPI^+^ cells/DAPI^+^ cells.

## 3 Results

### 3.1 Cryopreservation of passaged OPCs

**Figure 1:**
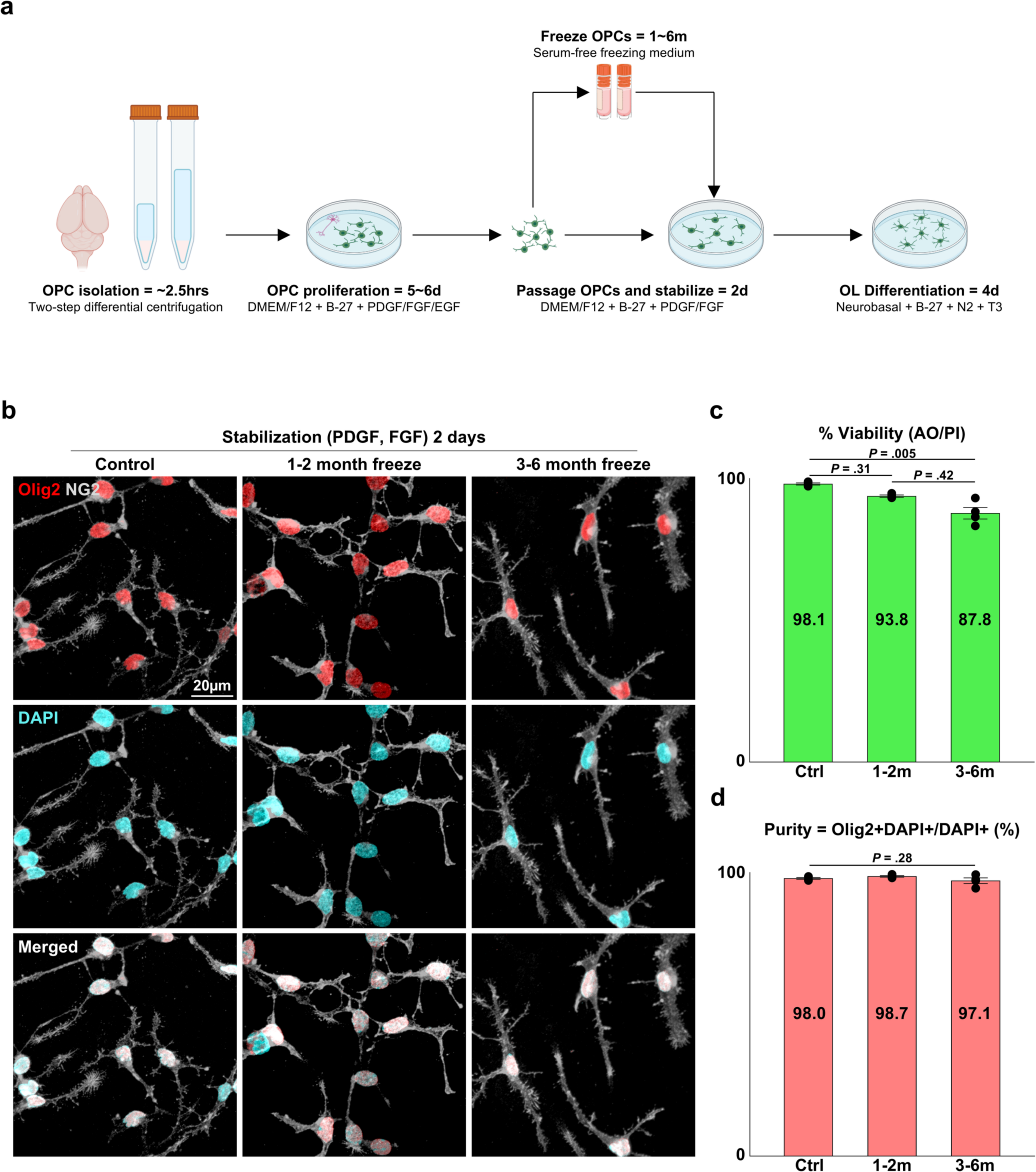
Viability and OL lineage purity of cryopreserved OPCs. **(A)** Illustration of the pipeline for primary OL culture and cryopreservation. **(B)** Representative images of control and cryopreserved OPCs after 2 days of stabilization. Immunostaining for NG2 and Olig2. **(C)** Graph of viability measured by a dual-fluorescence cell counter utilizing AO/PI (*n* = 4 biologically independent experiments per group, with three experimental groups. Statistical analysis was performed using a two-sided Kruskal–Wallis test. Data are presented as mean ± SEM). **(D)** Graph of OL lineage purity measured as Olig2^+^ DAPI^+^ OPCs/DAPI^+^ total cells (%) (*n* = 4 biologically independent experiments per group, with three experimental groups. For each group, 10 images were analyzed, with approximately 75 DAPI^+^ cells per image. Statistical analysis was performed using a two-sided Kruskal–Wallis test. Data are presented as mean ± SEM). OPC, oligodendrocyte progenitor cell; PDGF, platelet-derived growth factor; FGF, fibroblast growth factor; EGF, epidermal growth factor; T3, thyroid hormone T3; AO/PI, acridine orange/propidium iodide.

In previous studies, the authors have demonstrated a method utilizing two-step differential centrifugation to isolate OPCs from P1 SD rats, which we have termed the “E3 (easy, efficient and effective) method” (Kim et al., 2024). After 5 days of proliferation, the isolated OPCs could be passaged and stabilized for an additional 2 days to yield OPC cultures of 98–99% purity (Kim et al., 2024). The cell count at the passaging step was 3–5 × 10^6^ OPCs/pup, and a substantial excess of passaged OPCs were unused and discarded. Cryopreservation was applied to make use of the excess OPCs (Figure 1A). The commercially available, serum-free cryopreservation medium CELLBANKER 2 was selected, as serum is known to influence the fate of OPCs towards type 2 astrocytes (Suzuki et al., 2017; Hu et al., 2010; Raff et al., 1983). A brief pilot study revealed that if combined with cooling rate control using isopropanol, passaged OPCs cryopreserved at −80°C for 1–2 months had a viability of 90–95% when measured with a dual-fluorescence cell counter utilizing AO/PI, and also appeared to successfully reproduce basic *in vitro* OL characteristics such as OPC proliferation and differentiation when observed with a brightfield microscope.

To confirm the implications that cryopreservation is a viable option for primary OPCs cultured through the E3 method, we devised experiments to validate the viability and purity of cryopreserved OPCs and the extent to which they could recapitulate OL functions observed *in vitro*, such as OPC proliferation, OL differentiation, and *in vitro* myelination. Three experiment groups were compared: (1) control = OPCs that were directly subcultured without freezing, (2) OPCs cryopreserved at −80°C for 1–2 months, (3) OPCs cryopreserved at −80°C for 3–6 months. When the viability of OPCs from each group was measured with a dual-fluorescence cell counter, the control group had a viability of ~98%, the 1–2 months cryopreservation group showed ~94%, and the 3–6 months cryopreservation group showed a viability of ~88%. The viability of cells in the control group was significantly different from cells in the 3–6 months cryopreservation group (Figure 1C) (*p* = 0.005, two-sided Kruskal–Wallis test with Dunn’s multiple comparison test, *n* = 4 independent experiments per group).

**Figure 2:**
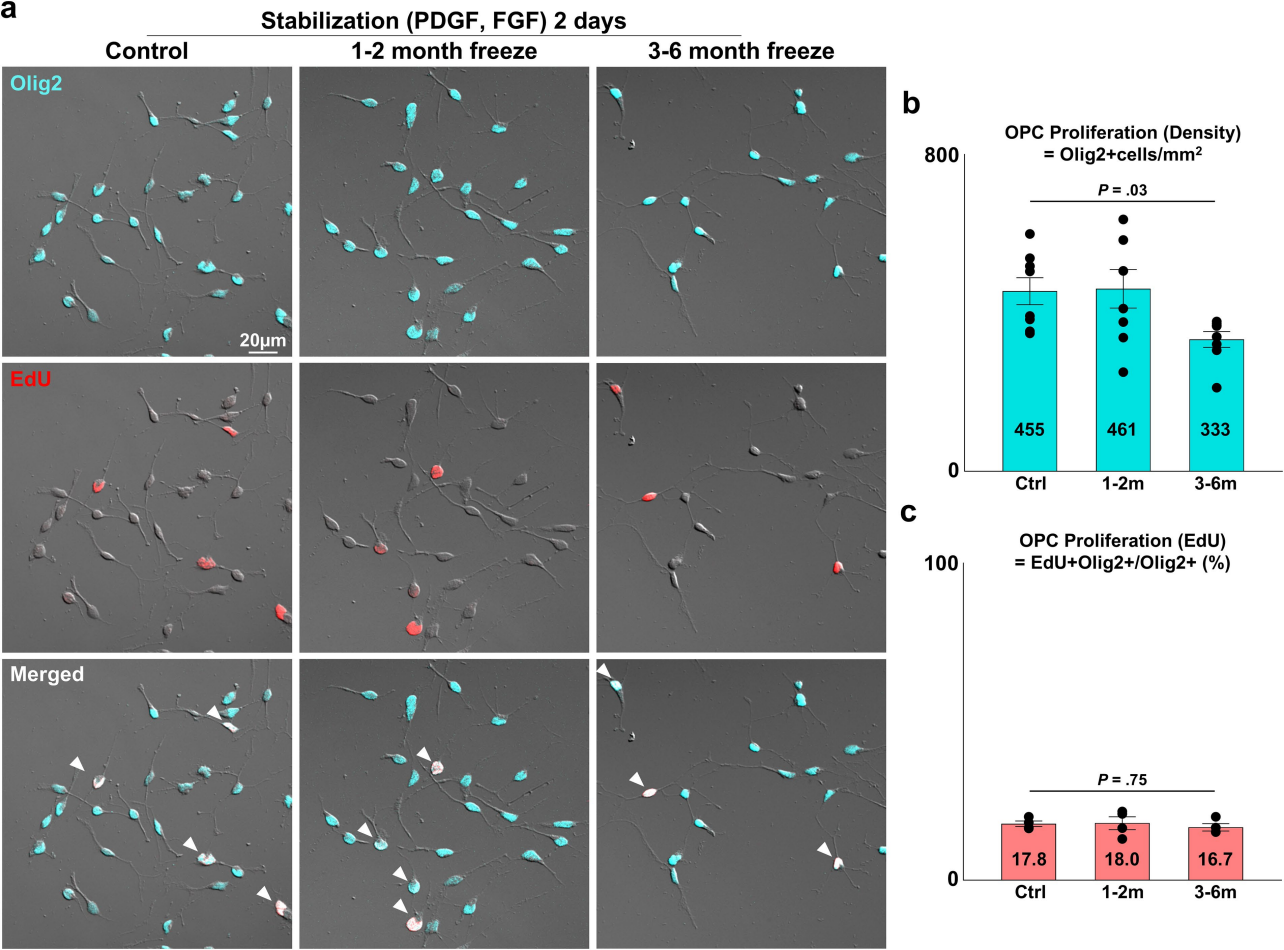
Proliferative capacity of cryopreserved OPCs. **(A)** Representative images of control and cryopreserved OPCs after 2 days of stabilization. Staining for Olig2, EdU. White arrowheads = proliferating OPCs positive for both Olig2 and EdU. **(B)** Graph of OPC density per mm^2^ measured as Olig2^+^ cells/mm^2^ (*n* = 8 biologically independent experiments per group, with three experimental groups. For each group, 16 images were analyzed, with approximately 75 Olig2^+^ cells per image. Statistical analysis was performed using a two-sided one-way analysis of variance (ANOVA). Data are presented as mean ± SEM). **(C)** Graph of OPC proliferation measured as EdU^+^ Olig2^+^ OPCs/Olig2^+^ total cells (%) (*n* = 4 biologically independent experiments per group, with three experimental groups. For each group, 16 images were analyzed, with approximately 75 Olig2^+^ cells per image. Statistical analysis was performed using a two-sided Kruskal–Wallis test. Data are presented as mean ± SEM). OPC, oligodendrocyte progenitor cell; PDGF, platelet-derived growth factor; FGF, fibroblast growth factor.

Another critical and frequently reported parameter used to evaluate the quality of OL cultures is the purity of OL lineage cells, as primary OL cultures are initially isolated from brain tissue that contains potential contaminants, such as neurons and other glial cells. We have documented 98–99% purity for the E3 method for OPCs stabilized for 2 days after passaging by quantifying the proportion of Olig2^+^ cells. In concordance with our past reports, all three experimental groups exhibited 97–99% purity (Figure 1D) (*p* = 0.24, two-sided Kruskal–Wallis test, *n* = 4 independent experiments per group), and the cells could be discerned as OPCs judged by their expression of NG2 and their bipolar morphology (Figures 1B, 2A).

### 3.2 Proliferative capacity of cryopreserved OPCs

After validating the viability and purity of cryopreserved OPCs, the three experimental groups were compared for OPC proliferation, an essential function of OPCs as progenitors of the OL lineage. The number of viable OPCs seeded was kept consistent for all three experiment groups. The seeded OPCs were maintained for 2 days in stabilization media containing PDGF-AA and FGF, two mitogens known to synergistically promote OPC proliferation and prevent differentiation into mature OLs (McKinnon et al., 1990; Baron et al., 2000; Hart et al., 1989; McKinnon et al., 1991). Proliferation was assessed through two analyses: (1) measurement of OPC cell density (=Olig2^+^ cells/mm^2^), (2) proportion of OPCs positive for the proliferation marker EdU (=EdU^+^ Olig2^+^/Olig2^+^ cells). The density of OPCs was ~450 OPCs/mm^2^ for the control and 1–2 month cryopreservation group, and 333 OPCs/mm^2^ in the 3–6 months cryopreservation group (Figure 2B) (*p* = 0.03, two-sided Kruskal–Wallis test, *n* = 8 independent experiments per group). The proportion of OPCs positive for EdU was approximately 17% for all groups (Figure 2C) (*p* = 0.75, two-sided Kruskal–Wallis test, *n* = 4 independent experiments per group).

### 3.3 Differentiation of cryopreserved OPCs into mature OLs

**Figure 3:**
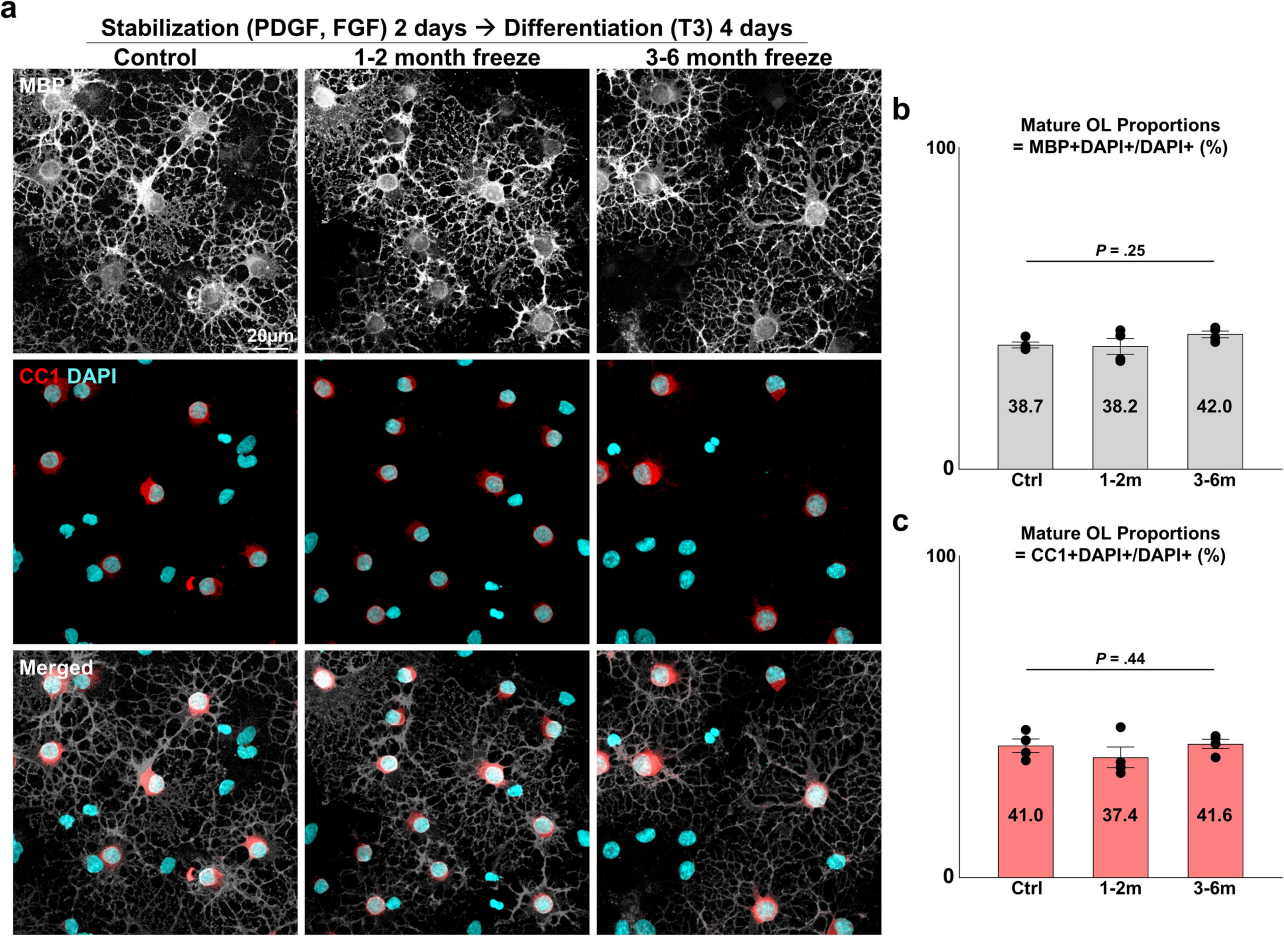
Differentiation of cryopreserved OPCs into mature OLs. **(A)** Representative images of control and cryopreserved OPCs after 2 days of stabilization, followed by 4 days of differentiation into mature OLs. Immunostaining for MBP, CC1. **(B)** Graph of mature OL proportions, measured as MBP^+^ DAPI^+^ mature OLs/DAPI^+^ total cells (*n* = 4 biologically independent experiments per group, with three experimental groups. For each group, 10 images were analyzed, with approximately 75 DAPI^+^ cells per image. Statistical analysis was performed using a two-sided Kruskal–Wallis test. Data are presented as mean ± SEM). **(C)** Graph of mature OL proportions, measured as CC1^+^ DAPI^+^ mature OLs/DAPI^+^ total cells (*n* = 4 biologically independent experiments per group, with three experimental groups. For each group, 10 images were analyzed, with approximately 75 DAPI^+^ cells per image. Statistical analysis was performed using a two-sided Kruskal–Wallis test. Data are presented as mean ± SEM). OPC, oligodendrocyte progenitor cell; OL, oligodendrocyte; MBP, myelin basic protein; PDGF, platelet-derived growth factor; FGF, fibroblast growth factor; T3, thyroid hormone T3.

**Figure 4:**
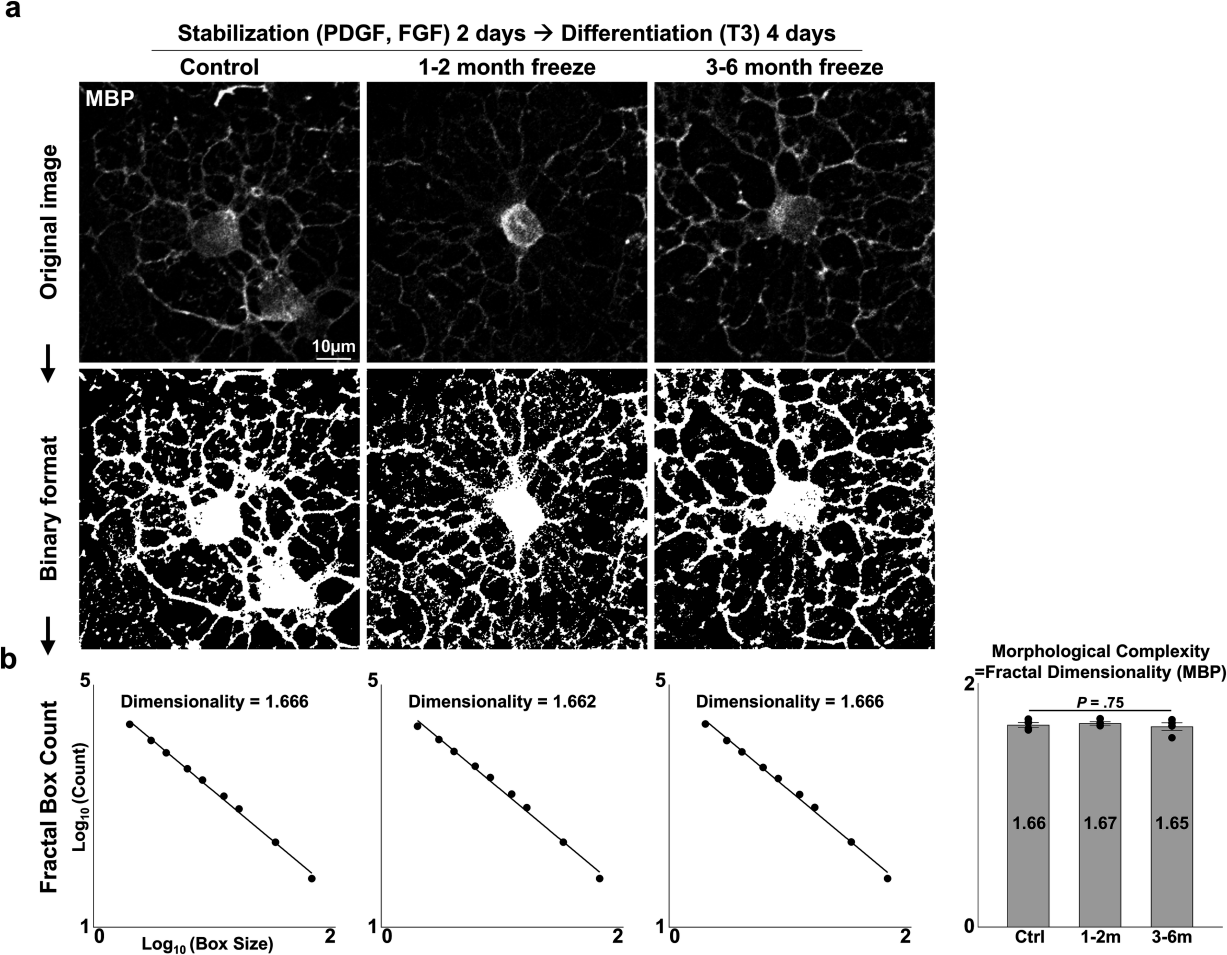
Morphological complexity of cryopreserved mature OLs. **(A)** Representative images of control and cryopreserved OPCs after 2 days of stabilization, followed by 4 days of differentiation into mature OLs. Immunostaining for MBP. **(B)** Measurement of fractal dimensionality by the fractal box counting method and graph of morphological complexity, measured as the fractal dimensionality of MBP-stained images of mature OLs (*n* = 4 biologically independent experiments per group, with three experimental groups. For each group, 40–80 images were analyzed, containing 1–2 MBP^+^ OLs per image. Statistical analysis was performed using a two-sided one-way analysis of variance (ANOVA). Data are presented as mean ± SEM). MBP, myelin basic protein; PDGF, platelet-derived growth factor; FGF, fibroblast growth factor; T3, thyroid hormone T3.

Another crucial function of the OL lineage is the differentiation of OPCs into mature, myelinating OLs. After 2 days of OPC stabilization, we induced differentiation with an NBM-based differentiation medium containing thyroid hormone T3, a well-established inducer of *in vitro* and *in vivo* OL differentiation (Barres et al., 1994). After 4 days of differentiation, APC-CC1^+^ and MBP^+^ mature OLs could be observed (Figure 3A). Complex, web-like myelin processes, which are characteristic of OLs cultured on two-dimensional surfaces, were evident in all experiment groups. The extent of maturation was evaluated with two parameters: (1) the proportion of mature OLs (=MBP^+^ DAPI^+^/DAPI^+^ cells, APC-CC1^+^ DAPI^+^/DAPI^+^ cells), (2) morphological complexity of MBP^+^ processes (=measurement of fractal dimensionality through the fractal box counting method in Fiji/ImageJ). Cryopreserved OPCs did not differ in terms of the proportion of cells that matured (Figures 3B,C) (*p* = 0.25 and 0.44, two-sided Kruskal–Wallis test, *n* = 4 independent experiments per group) nor in terms of morphological complexity (Figures 4A,B) (*p* = 0.75, one-way ANOVA, two-sided, *n* = 4 independent experiments per group). Thus, we concluded that cryopreserved OPCs could adequately develop into mature OLs.

### 3.4 *In vitro* myelination of cryopreserved OLs

**Figure 5:**
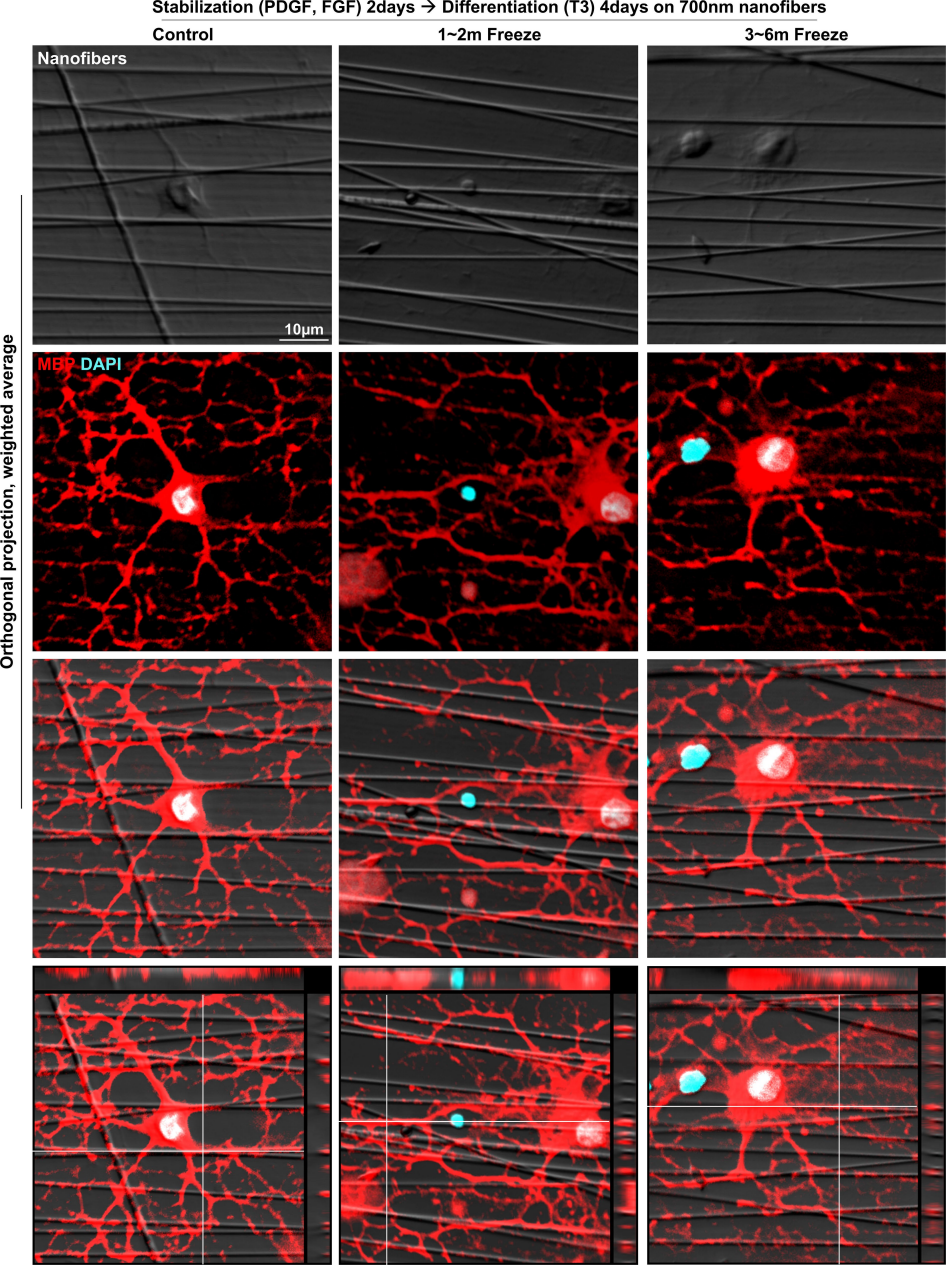
*In vitro* myelination of cryopreserved mature OLs. Representative images of control and cryopreserved OPCs cultured on aligned nanofibers, for 2 days of stabilization followed by 4 days of differentiation into mature OLs. Immunostaining for MBP (Z-stack images, five slices spaced 1 μm apart). MBP, myelin basic protein; PDGF, platelet-derived growth factor; FGF, fibroblast growth factor; T3, thyroid hormone T3.

After confirmation of successful OL differentiation, the last function to be evaluated was myelination, the OL characteristic directly related to saltatory conduction. It has been demonstrated in previous studies that myelination can be achieved *in vitro* by culturing primary OLs on aligned nanofibers of appropriate caliber, mimicking axons in the white matter of the brain (Bechler, 2019). For *in vitro* myelination, OPCs were seeded onto inserts with aligned polycaprolactone (PCL) nanofibers of ~700 nm diameter, stabilized for 2 days, and subsequently differentiated for 4 days. Upon immunofluorescent staining with MBP, instances of MBP^+^ myelin processes enwrapping the fibers could be observed in both control and cryopreserved groups, indicating that cryopreserved OLs were able to form myelin sheath-like structures around axon-mimicking structures (Figure 5). In summary, cryopreserved OPCs could recapitulate *in vitro* OL functions in a similar manner as non-frozen controls.

## 4 Discussion

*In vitro*, primary OL culture has been vital to research on OLs and myelination. The OL lineage comprises various stages that serve different functions: OPCs, pre-OLs, immature OLs, and mature OLs. *In vitro* OL culture models generally share a common process, starting with the acquisition of OPCs and ending with their terminal differentiation into mature OLs. This common process allows researchers to easily separate and focus on specific stages and functions of interest, which is much more complicated in an *in vivo* system where all stages and functions are simultaneously at play and intertwined. To improve efficiency and simplicity, an aspect where primary culture models generally underperform compared to immortalized cell lines, the authors have recently published the E3 method for primary OL culture with which researchers can acquire large numbers of OPCs with minimal technical and material requirements (Kim et al., 2024). To make use of excess OPCs that would otherwise be discarded, we have established a method to cryopreserve primary OPCs cultured through the E3 method. Previous reports on the usage of cryopreserved primary OLs have not scrutinized the functional validity of the frozen OLs, and utilize cells cultured from stem cells through the “oligosphere” method, which, albeit significant strengths, especially in terms of enabling the acquisition of primary human OLs, has the drawback of an extensive culture period (Larsen et al., 2008; Espinosa-Jeffrey et al., 2009). In this report, we demonstrate the process for cryopreservation of cultured rat OPCs and show the degree to which they could recapitulate *in vitro* OL functions such as OPC proliferation, OL differentiation, and *in vitro* myelination. Through the cryopreservation of primary OLs, the effort required for utilizing primary OLs for research could be significantly reduced. Additionally, the magnitude of animal sacrifice, an undesired yet unavoidable component of primary cell culture, could be substantially reduced with the cryopreservation and usage of primary cells.

The first step in validating cryopreserved OPCs as a viable *in vitro* cell culture model was to confirm the viability of the frozen OPCs and ensure that no changes occurred in cell type, as OPCs are known to be bipotential and capable of differentiating into type 2 astrocytes. Despite a drop in the percentage of live cells for cryopreserved OPCs, they indifferently formed OPC cultures of 97–99 purity when the designated number of live OPCs was seeded, which is consistent with our previous report on OPCs at this phase in the E3 culture method.

The density of the OPC cultures after 2 days of stabilization in proliferation media containing PDGF and FGF was roughly 450 OPCs/mm^2^ for both control and OPCs cryopreserved for 1–2 months. OPCs frozen for 3–6 months showed a lower cell density of 333 OPCs/mm^2^. This was an approximately three to four-fold increase from the initial seeding density of 100 OPCs/mm^2^ (=1 × 10^4^ OPCs/cm^2^). Despite differences in cell density, ~17% of OPCs in all groups were positive for the cell cycle marker EdU at the end of the two-day stabilization period. As EdU is uptaken by cells specifically in the DNA synthesis phase of the cell cycle (Harris et al., 2018), this arguably indicates that more than 17% of OPCs, control and frozen alike, maintained capacity for cell division after the 2 days of stabilization. Considering the consistency of EdU uptake and the decrease in cell viability for the 3–6 months group, the most plausible explanation for the decrease in cell density may be a delay in the initiation of proliferation, or cell damage.

After proliferation, cryopreserved OPCs could successfully differentiate into mature OLs. The proportion of OPCs which differentiated into mature OLs, and the morphological robustness of the mature OLs, was comparable between control and cryopreserved OPCs. It was also confirmed that cryopreserved OLs could form myelin sheath-like structures colocalizing with aligned nanofibers mimicking axon structure, suggestive of myelination. Thus, we could validate that cryopreservation did not negatively impact the maturation process or the primary function of myelin sheath formation. This allows cryopreserved OLs to be utilized in experiments or applications that require functionally robust OLs. One example of this would be the transplantation of OLs, which has been shown to be effective in initiating remyelination in animal models of demyelination (Li et al., 2021; Wang et al., 2020; Xu et al., 2015; Wu et al., 2012; Wang et al., 2021). Storage and usage of cryopreserved OPCs will enable and greatly improve the practicality of OPC transplantation, which may further benefit from methods that allow for longer periods of cryopreservation. Methods to cultivate OPC/OLs from different organisms, which are efficient and cryopreservation, will further expand the applications for cryopreserved OLs.

During the validation of cryopreserved OPCs/OLs, we discovered a decrease in cell density in the 3–6 months cryopreservation group after proliferation. While the OPCs in the 3–6 months cryopreservation group were still capable of completing the subsequent functions of differentiation and myelin sheath-like structure formation, this result shows that the cryopreserved OLs, after long term storage at −80°C, were not immaculately the same as the controls. Generally, the use of −196°C liquid nitrogen (LN) storage is recommended for cell cryopreservation, especially if considering long term storage. In this study, a −80°C mechanical freezer was used. LN storage, when well-performed, can halt the formation of intracellular ice crystals, allowing for a near-indefinite storage of cells (Ramos et al., 2014). Primary OPCs are not propagatable in the same manner as immortalized cell lines, and the depletion of stored cells occurs much faster, placing emphasis on short term cryopreservation at −80°C. We have demonstrated that primary OPCs can be cryopreserved at −80°C for up to 2 months, without significant alterations in viability and canonical OL functions. Cryoprotectants to allow for long-term storage at −80°C are being developed, which may alleviate the downsides associated with −80°C storage (Zhou et al., 2024). In circumstances where mass production and long term storage is necessary, such as industries, LN storage may be an appealing option, although this has not been validated in this study.

In summary, the present study illustrates the process of cryopreserving cultured primary OPCs and the functional validity of frozen OPCs and OLs. The availability of cryopreserved primary OPCs may enable and greatly improve the efficiency of experiments and applications requiring functional primary OLs, and help reduce the inevitable animal sacrifice associated with primary culture.

## Data Availability

The original contributions presented in the study are included in the article/supplementary material, further inquiries can be directed to the corresponding author.
